# Mild Two-Step Thermochemical Recovery of Clean Glass Fibers from Wind-Blade GFRP

**DOI:** 10.3390/polym17243344

**Published:** 2025-12-18

**Authors:** AbdulAziz AlGhamdi, Imtiaz Ali, Salman Raza Naqvi

**Affiliations:** 1 Department of Chemical Engineering, College of Engineering, Imam Mohammad Ibn Saud Islamic University (IMSIU), Riyadh 11564, Saudi Arabia; 2 Department of Chemical Engineering, College of Engineering, Prince Mohammad Bin Fahd University, Al Khobar 34754, Saudi Arabia; iali1@pmu.edu.sa; 3 Department of Engineering and Chemical Sciences, Karlstad University, 651 88 Karlstad, Sweden

**Keywords:** thermochemical recycling, glass fiber-reinforced polymer (GFRP), wind turbine blade waste, two-step pyrolysis–oxidation

## Abstract

End-of-life wind turbine blade accumulation is a growing global materials management problem and current industrial recycling routes for glass fiber-reinforced polymer composites remain limited in material recovery value. There is limited understanding on how to recover clean glass fibers while keeping thermal exposure and energy input low, and existing studies have not quantified whether very short isothermal thermal residence can still result in complete matrix removal. The hypothesis of this study is that a mild two-step thermochemical sequence can recover clean glass fibers at lower temperature and near zero isothermal dwell if pyrolysis and oxidation are separated. We used wind-blade epoxy-based GFRP in a step-batch reactor and combined TGA-based thermodynamic mapping, short pyrolysis at 425 °C, and mild oxidation at 475 °C with controlled dwell from zero to thirty minutes. We applied model-free kinetics and machine learning methods to quantify activation energy trends as a function of conversion. The thermal treatment of 425 °C for zero minutes in nitrogen, followed by 475 °C for fifteen minutes in air, resulted in mechanically sound, visually clean white fibers. These fibers retained 76% of the original tensile strength and 88% of the Young’s modulus, which indicates the potential for energy-efficient GFRP recycling. The activation energy was found to be approximately 120 to 180 kJ mol^−1^. These findings demonstrate energy lean recycling potential for GFRP and can inform future industrial scale thermochemical designs.

## 1. Introduction

Fiber-reinforced polymer (FRP) composites, particularly glass fiber-reinforced polymers (GFRPs), have become indispensable in various industries, including transportation, energy, marine, construction, and infrastructure, as they combine high specific strength and stiffness with corrosion resistance, fatigue resistance, and design flexibility at a competitive cost. Their quick adoption stems from mature manufacturing techniques, including pultrusion, infusion, and molding, along with matrix chemistries primarily consisting of thermosets, such as epoxies, unsaturated polyesters, and vinyl esters. The thermosets make an excellent bond between fibers and the matrix as well as have long-lasting reliability in severe conditions; however, the cross-linking provides an insoluble and non-infusible nature to thermosets after they cure and thus cannot be recycled via traditional re-manufacturing techniques, therefore making EOL management of these composite products such as boat and auto parts, wind turbine blades, etc., which are now being manufactured on a larger scale than ever before, even more problematic with landfill and basic incineration options being limited or the less desirable options. Recent comprehensive reviews synthesize this dual reality: booming deployment of FRP structures and an urgent need for scalable, higher-value recycling and waste-management solutions for thermoset-based systems. They also document the surge in research on reprocessable thermosets (e.g., vitrimer chemistries) and recyclable thermoplastic-matrix FRPs as complementary long-term strategies [1].

Within the clean-energy sector, wind power exemplifies both the opportunities and the challenges ahead. Global wind capacity surpassed 900 GW by 2022, with blades typically retiring after 20 to 25 years. A single 1 MW turbine can yield >10 t of blade waste at EoL, and cumulative waste blades may reach ~43 Mt by 2050, about 40% in China. Because commercial blades are predominantly GFRP, comprising ~60–70 wt% glass fibers bound in a 30–40 wt% thermoset resin, closing the loop on glass fiber and matrix resources is a compelling circular-economy target [2].

Conventional options each have limitations. Mechanical size reduction generates short, damaged fibers and mixed powders that typically feed only downgraded applications at modest loading levels (often ≤10%), while posing dust and handling hazards. Chemical solvolysis can preserve fiber properties more effectively, and sub- or supercritical media (e.g., water, alcohols) continue to mature; however, the need for aggressive reagents, elevated pressures/temperatures, and pre-shredding (which shortens fibers) impairs both economic and environmental feasibility. Thermal routes include incineration, fluidized-bed low-temperature oxidation, gasification, and pyrolysis. Of these, fluidized-bed processes can handle mixed waste but often erode the tensile strength of glass fibers (e.g., a ~50% loss observed in early studies), undermining their reuse value; incineration recovers only heat and sacrifices materials. Collectively, reviews conclude that a scalable, economically sustainable solution for thermoset-FRP waste remains elusive, spurring research on both improved recycling processes now and on redesigning matrices (e.g., vitrimers) for future circularity [1,3].

Against this background, pyrolysis, the thermal decomposition of the organic matrix in an inert atmosphere, has become a promising short-term method for recycling and resource recovery of FRP. In pyrolysis, the cured polymer network is cleaved to yield a gas (often energy-rich), a condensable oil fraction (which can serve as fuel or chemical feedstock), and a solid residue comprising fibers, fillers, and char. Classic and contemporary studies on glass–polyester and glass–epoxy wastes demonstrate that optimized pyrolysis can: (i) remove matrix while limiting glass fiber damage; (ii) generate oil streams enriched in aromatics (e.g., styrene) and oxygenates (e.g., phthalic anhydride) that could be valorized; and (iii) deliver gases (CO, CO_2_ with H_2_/CH_4_/C_2_–C_4_) that can help meet the process energy demand. For example, fixed-bed pyrolysis of a commercial GRP at 450 °C produced gas dominated by CO+CO_2_ (>75 vol%), with oil containing valuable aromatic/oxygenated species; recovered fibers could replace up to ~20 wt% of virgin glass fiber in dough-molding compounds [4].

The technical case for pyrolysis has only strengthened as feedstocks shift from general GRP to large EoL blade sections and manufacturing scrap. A 2018 critical review concluded that pyrolysis enables reuse of all waste components (fibers, oils, gases), and cataloged process parameters and outcomes across reactors (fixed-bed, auger, fluidized), temperatures (~300–700 °C), residence times, and post-treatments (oxidative “polishing” to remove residual char). It also documented typical product distributions (e.g., a slow pyrolysis case at 550 °C: ~24 wt% oil, 8 wt% gas, and 68 wt% solids). It highlighted that post-pyrolysis oxidation can render fibers “clean” for downstream compounding or glass–ceramic valorization. Importantly, that review contrasted pyrolysis favorably with low-temperature combustion, which recovered fibers but at the expense of severe strength loss in glass fibers [3].

Recent mechanistic work has deepened our understanding of epoxy-matrix degradation in GFRPs and suggested pathways to higher pyrolysis efficiency. Combined experimental and density-functional-theory (DFT) analyses on retired wind turbine blade materials report mass losses between ~290 and 500 °C with an apparent activation energy ≈ 170 kJ mol^−1^, consistent with free-radical scission of C–O bonds and formation of intermediates that recombine into characteristic products (e.g., bisphenol-A, epoxides, propylene). Intriguingly, the presence of glass fibers (SiO_2_) lowers C–O bond orders and accelerates matrix dissociation. Process-wise, a synergistic pyrolysis-then-oxidation sequence has achieved a pyrolysis efficiency of ~89.5% and removed organic residues exceeding 98%, yielding surface-clean fibers suitable for reuse. These insights underpin reactor and recipe design (temperature profiles, residence time, carrier flow, staged oxidation) tailored to composite architecture and desired fiber quality [5].

The potential for pyrolysis remains unproven for consistently generating high-value secondary products from large volumes of recovered glass fibers (rGFs).

- Thermal and mechanical stresses damage fibers. As a result, without exact control of variables including temperature limits, oxygen exposure, and residence times, post-processing treatment of the recycled glass fibers (rGFs), in addition to their length and the extent of surface irregularities, can reduce the original properties of the fibers. Additionally, deterioration of any original sized coatings applied to the glass fibers can negatively affect bonding between the recycled fibers and other components in the new composite material. Therefore, it is important to develop sizing techniques that are specific to the rGF surface [3].
- The core of the blade laminate includes foam, wood, and other adhesives/minerals, such as calcium carbonate (CaCO_3_). Additionally, the presence of various volatiles during processing creates additional difficulties in managing the removal of these volatiles and inorganic fouling of the blades. It also creates additional complexity in recovering oil and gas, as well as maintaining the quality of the glass fibers, thus making process control and residue management even more complicated. Traditional fixed-bed studies have been successful in developing process conditions that do not decompose fillers; however, today’s multiple-component blade laminates require that this selectivity be extended to the entire laminated assembly [1].
- Pyrolysis oil is a potential fuel source; however, the greater value in the chemical stream (e.g., styrene streams, aromatic building blocks) will increase the overall economic viability through additional process steps (upgrading, separation, and speciation). A gas-based energy recovery system should be included to reduce utility requirements. Examples of utilizing resin-derived streams in an open loop (e.g., superplasticizer production from an epoxy degradative solution) demonstrate how new value chains can be developed on a larger scale [1,6].
- The reviews of individual blades are based on the fact that the grade of fibers after recycling determines their suitability for secondary uses. It is necessary to develop standards for grading and fractioning recycled glass fibers (rGFs), establish standardized re-sizing processes, and create rules that relate the properties of rGFs to specific product types (e.g., reinforcing concrete, thermoplastics compounding) to facilitate continued market expansion.

Pyrolysis presents a viable solution for managing thermoset-GFRP waste by utilizing suitable materials and recovery techniques as the industry transitions to recyclable matrices. Years of research, ranging from basic fixed-bed experiments on glass–polyester to advanced blade-tailored, mechanism-based processes, show that rGFs can be recovered in a functional form and resin-based streams can be valorized through optimized thermal treatments and post-oxidation cleaning. The remaining research priorities are clear: (i) quantifying and improving rGF performance via gentle thermal histories and effective re-sizing; (ii) integrating product upgrading to capture chemical value; (iii) managing multi-material feedstocks; and (iv) codifying quality standards that connect recycled outputs to robust markets. Addressing these will move pyrolysis from “promising” to “bankable” in the FRP circular economy while next-generation materials (e.g., vitrimers) reduce tomorrow’s recycling burden by design.

Table 1 presents the literature analysis of the thermochemical recycling of glass fiber-reinforced composites from 2018 to 2025. Recent reviews position pyrolysis as a leading near-term route for EoL GFRP/CFRC because it can treat large, heterogeneous parts and enables subsequent fiber re-sizing for secondary composites [3]. LCA on an advanced two-step variant (primary pyrolysis followed by high-T cracking of volatiles) reports improved environmental performance compared to one-reactor schemes, while reclaiming fibers and producing valuable syngas-range gases [3]. Thermogravimetry/kinetics on GFRP indicate a significant mass loss between ~300 and 500 °C, with activation energies consistent with epoxy network scission. The product gases and oils can supply process heat or be upgraded [7]. Engineering the vapor phase boosts value: Cracking pyrolysis volatiles at ~900 °C shifts liquids to H_2_, CO, CH_4_, and C_2_, aligning with syngas targets [8]. Fiber quality improves with sequenced pyrolysis → mild oxidation → surface treatment; partial oxidation plus brief alkaline etching has restored rGF tensile strength (up to ~2× vs. only-pyrolyzed fibers) [9].

**Figure 1:**
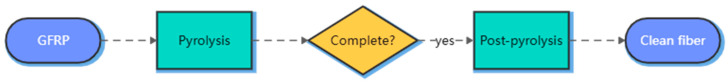
Schematic view of the research plan.

In this work, we propose a mild thermochemical pathway for the selective recovery of glass fiber from wind-blade glass fiber-reinforced polymer waste (Figure 1). The scientific aim of the work is to define the lowest temperature and shortest exposure times that still enable complete matrix removal without compromising the intrinsic mechanical integrity of the recovered glass fibers. The study combines thermogravimetric analysis, step-batch reactor trials, kinetic modeling, and machine learning to quantify decomposition regimes and the evolution of activation energy. This combination provides a strong empirical and mechanistic basis for determining gentle operating boundaries for both pyrolysis under nitrogen and post-pyrolysis oxidation under air. The novelty lies in defining and validating a low-temperature two-step operational window that separates pyrolysis from oxidation while minimizing isothermal dwell time. The identified conditions of 425 °C for pyrolysis followed by 475 °C for oxidation, with no pyrolysis dwell and only 15 min of oxidation dwell, challenge the conventional assumption that temperatures above 500 °C or long residence times are required. This enables cleaner fibers with significantly reduced energy input. Key findings include a single dominant degradation region between 300 and 500 °C, a characteristic activation energy trend from about 170 kJ mol^−1^ to 120 kJ mol^−1^ and back to 180 kJ mol^−1^, and the strong predictive capability of the classification and regression tree model, which achieved an R^2^ of 0.968 for activation energy prediction. Together, these results provide a data-driven path toward industrially practical and energy-efficient glass fiber reclamation.

## 2. Materials and Methods

### 2.1. Sample Collection

GFRP, sourced from wind turbine blades, is a sandwich structure of polymeric foam with fiberglass panels in an epoxy matrix. The material is cut into 23 mm × 30 mm × 150 mm pieces using a diamond cutter.

### 2.2. Thermodynamic Analysis

Thermogravimetric (TGA) and differential thermogravimetric (DTG) measurements were performed on a TA Instruments TGA550 (TA Instruments, New Castle, DE, USA) to resolve the thermodynamic decomposition of wind-blade GFRP under oxidative (air) atmospheres. Samples were heated from ambient to 900 °C at controlled rates of 5, 10, 20, and 40 °C min^−1^. The combined TGA/DTG outputs define conservative setpoints that informed reactor trials, minimizing isothermal dwell while achieving full matrix removal by separating polymer volatilization (pyrolysis) from char burn-off (post-pyrolysis).

### 2.3. Experimental Setup

**Figure 2:**
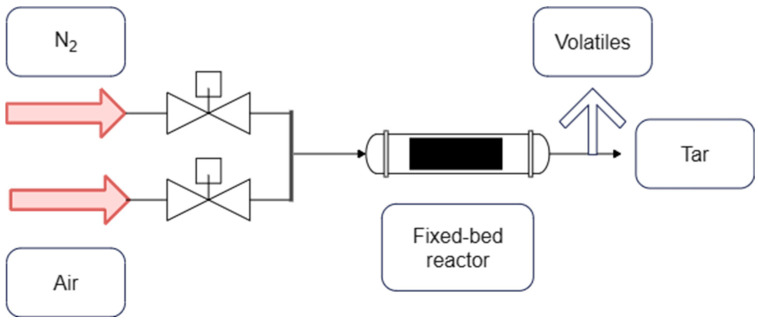
Schematic view of the experimental setup [15].

A laboratory step-batch reactor was employed for thermochemical treatment, and its schematic is shown in Figure 2. The oven is a horizontal, cylindrical furnace (inner tube: 40 mm i.d., 600 mm heated length) housed within a 240 mm o.d. shell containing the electric heating elements and insulation. Feed and purge gases are selected via two manual valves: one connected to nitrogen for inert pyrolysis and the other to oxygen/air for post-pyrolysis oxidation. Downstream of the furnace, a fume extractor and a dedicated tar outlet manage condensable vapors and off-gases, ensuring the clear separation of solid residue (fibers and char) from volatile products. Temperature control is achieved with a thermostat; spatial/temporal variations in the setpoint are within ±10 °C. Process conditions were explored in four systematic series as mentioned in our previous short study (Table 2) [15]: (i) Effect of temperature during pyrolysis (N_2_)—the setpoint was increased from 350 °C to 450 °C at a fixed isothermal dwell of 30 min to identify the lowest temperature yielding complete matrix decomposition. (ii) Effect of residence time during pyrolysis (N_2_)—at the selected “mild” temperature of 425 °C, the isothermal dwell was varied (0, 15, 30 min) to minimize thermal exposure while achieving complete pyrolysis. (iii) Effect of temperature during post-pyrolysis (air/oxygen)—material pre-pyrolyzed at 425 °C for 30 min was oxidized at 400, 450, 475, and 500 °C for 30 min to remove residual char and clean fiber surfaces. (iv) Effect of residence time during post-pyrolysis (air/oxygen)—at the identified mild oxidation temperature of 475 °C, the dwell time was varied (0, 15, 30 min) to define the minimum exposure required for visually clean fibers.

These stepwise campaigns converged on a conservative, low-temperature window for both stages. Throughout, “residence time” denotes only the isothermal dwell at the target setpoint. The oven heat-up time (~10 min) and the oven cool-down time (~300 min) were removed from the study. The furnace design does not allow for the insertion of samples at the same temperature as the furnace’s setpoint, nor can samples be removed from the furnace at high temperatures. Thus, when reporting “0 min” for a dwell time, it means the sample experienced only controlled heating to the desired temperature setpoint, followed by a controlled cool-down, with no intended isothermal dwell time. This method was used uniformly throughout all experimental series. In addition to providing a broad range of temperature and dwell times, the datasets generated during pyrolysis (N_2_) and post-pyrolysis oxidation (air/oxygen) collectively yielded the least severe conditions that produced clean, white fibers and avoided excessive thermal exposure.

### 2.4. Kinetic Analysis

The fundamental rate equation for solid-state degradation is expressed as follows [16,17]:

(1) $$\frac{d\alpha }{dt}=Aexp\left(\frac{-E_{a}}{RT}\right)f(\alpha )$$

where *A* is the pre-exponential factor, *E_a_* is the apparent activation energy, *T* is the absolute temperature, R is the ideal gas constant, and *f*(*α*) is the reaction model.

#### 2.4.1. Model-Free Kinetic Analysis

The apparent activation energy was determined using the Friedman (FR), Kissinger–Akahira–Sunose (KAS) and Ozawa–Flynn–Wall (OFW) methods:

(2) $$\mathrm{FR}:\;ln\left(\beta \frac{d\alpha }{dT}\right)=ln[Af(\alpha )]-\frac{E_{a}}{RT}$$

(3) $$\mathrm{KAS}:\;ln\left(\frac{\beta }{T^{2}}\right)=\mathrm{Const}-\frac{E_{a}}{RT}$$

(4) $$\mathrm{OFW}:\;ln(\beta )=\mathrm{Const}-1.052\frac{E_{a}}{RT}$$

The Advanced Vyazovkin method, a non-linear integral method, was also applied for comparative analysis.

#### 2.4.2. Model-Fitting Kinetic Analysis

A model-fitting approach using a generalized reaction model was used where a combined kinetic plot was constructed using data from all heating rates to visualize the global reaction behavior.

### 2.5. Machine Learning

The classification and regression tree (CRT) algorithm, along with other machine learning models, was used to predict the activation energy (*E_a_*) derived from the Friedman method. The input parameters were conversion (*α*), temperature (*T*), and heating rate (*β*), with *E_a_* as the target output [18]. A dataset was constructed from TGA data acquired at multiple heating rates. The performance of Artificial Neural Networks (MLPs), CRT, Boosted Regression Trees (BRTs), and Multivariate Adaptive Regression Splines (MARS) was compared. MLP is a multilayer perceptron with an architecture optimized via an automated network search. CRT is a decision tree-based regression model. BRT is an ensemble model that sequentially and iteratively builds multiple trees. MARS is a flexible model that captures non-linear relationships using piecewise linear basis functions. The dataset comprised 363 data points extracted from TGA experiments. The data was split into training (70%), validation (15%), and testing (15%) sets using random sampling. All machine learning models were implemented using the TIBCO Statistica 13.5. The optimization process for each algorithm was as follows: the MLP architecture was optimized to a 3-5-1 structure via an automated network search. For the CRT, pruning determined an optimal tree depth of 15. The BRT model was configured with 100 trees and a learning rate of 0.1, parameters were selected through a grid search. Finally, the MARS model employed 11 basis functions, chosen to minimize Generalized Cross-Validation (GCV) error. Model performance was evaluated using the coefficient of determination (R^2^), mean square error (MSE), root mean square error (RMSE), and mean absolute error (MAE).

## 3. Results

### 3.1. TGA

**Figure 3:**
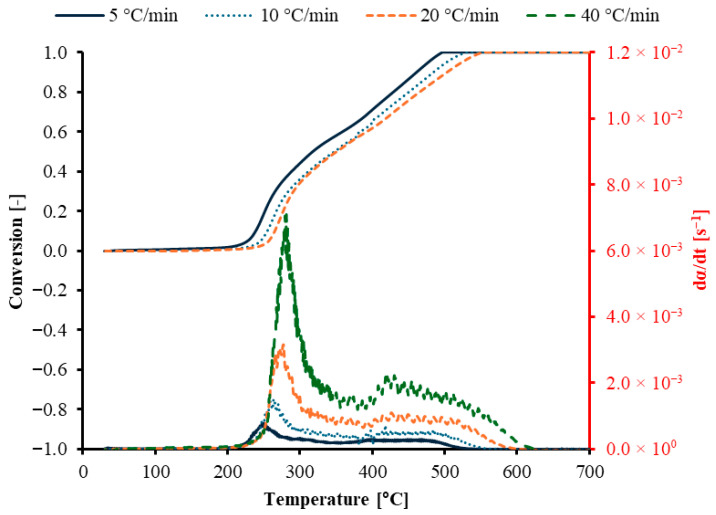
(D)TG curves of GFRC combustion at different heating rates.

The (D)TG curves at different heating rates are shown in Figure 3. This figure presents thermogravimetric analysis of a GFRC sample under four heating rates of 5, 10, 20, and 40 °C/min. The conversion curves show that thermal decomposition begins around 200 °C under all heating rates: the conversion rate increases and shifts to higher temperatures as the heating rate increases. The strongest peak in the conversion rate occurs at 40 °C/min, indicating that a high heating rate accelerates devolatilization and reduces heat-transfer limitations. A higher heating rate shifts decomposition to higher temperatures and increases the instantaneous rate, although the final conversion limit remains similar. This information is important when selecting reactor operating conditions and when fitting kinetic models from thermogravimetric data. Yousef et al. (2021) used thermogravimetric analysis of milled glass fiber-reinforced epoxy resin composite (GFRC) samples [19]. This study indicated that the milled GFRC samples decomposed in three successive stages, exhibiting similar features across different heating rates but with varying weight loss and decomposition zone positions. Similarly, Qiao et al. (2020) studied the degradation of glass fiber-reinforced plastic under inert conditions [20]. The first stage of weight loss occurs between 290 and 460 °C, followed by the second stage from 460 to 1000 °C. These two stages result from the pyrolysis of the epoxy resin matrix, while the glass fiber remains undecomposed. Recently, Yousef et al. (2024) analyzed the thermal decomposition of waste wind turbine blades (WTBs) and their components, including resin and fiber [21]. The analysis indicated that the primary decomposition reaction for WTBs occurred between 350 and 490 °C. This temperature range is crucial for understanding the material’s thermal stability and breakdown behavior. All these studies are in good agreement with this study.

### 3.2. Pyrolysis and Post-Pyrolysis

**Figure 4:**
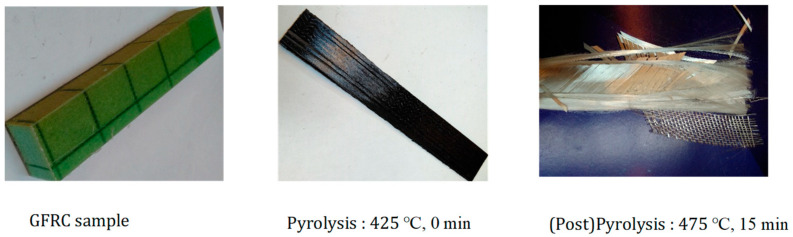
Sample treatment during pyrolysis and post-pyrolysis process.

Figure 4 shows three stages in the thermal treatment of a glass fiber-reinforced composite. The first image presents the intact composite before thermal treatment. The material still contains its polymer matrix, which keeps the glass fibers embedded and mechanically bonded. Its geometry remains uniform, and the surface is smooth and coherent. The second image shows the same composite after thermal exposure at 425 °C for zero-minute residence time. At this temperature, the polymer matrix immediately carbonizes at the surface, turning dark. There is no time for full matrix degradation. The structure, therefore, remains bonded, but the first chemical changes have already begun. This intermediate stage shows the onset of pyrolytic reactions, including devolatilization, partial carbonization, and very early softening events under oxygen-free conditions. The third image shows the post-pyrolysis (oxidation) condition at 475 °C for fifteen minutes. At this stage, nearly all the matrix has decomposed and pyrolyzed into gas and condensable fractions. The polymer that remained at 425 °C has now fully degraded, releasing the inorganic glass reinforcement fibers. These fibers appear white and clean because the organic carbon-rich residues have been removed. The fibers separate, delaminate, and lose structural adhesion because the matrix that originally provided mechanical transfer between fibers no longer exists. This final state represents successful selective reclamation of glass fibers through time and temperature-controlled pyrolysis. The complete removal of polymer is important for recovering fibers for further reuse, quality control in recycling, and material circularity studies in composite waste treatment research. The surface morphology and cleanliness of the recovered fibers under these conditions were previously confirmed by scanning electron microscopy, which showed complete resin removal and intact fiber surfaces, with no evidence of pitting or thermal erosion [15]. Zhang et al. (2024) studied the pyrolysis process and product features of glass fiber-reinforced composites [22]. They found that approximately 450 °C is the optimal pyrolysis temperature, at which fibers retain greater strength and oil yield is maximized.

### 3.3. Mechanical Characteristics of Recovered Glass Fibers

The mechanical properties of the recovered glass fibers (Table 3) were evaluated by measuring tensile strength and Young’s modulus, in accordance with the standardized single-filament tensile testing procedure [23]. The recycled fibers, produced utilizing the optimized mild two-step conditions (425 °C pyrolysis with 0 min dwell, succeeded by 475 °C oxidation for 15 min), exhibited a tensile strength of 1.52 GPa, which is a 24% reduction compared to virgin E-glass fibers at approximately 2.0 GPa; the Young’s modulus was 62 GPa, reflecting a decrease of roughly 12% from the typical 70 GPa of virgin fibers.

Regarding the context, previous pyrolysis-only processes exhibited greater degradation: Giorgini et al. (2016) reported 1.02 GPa (−50%) with a fixed-bed reactor [24], while Onwudili et al. (2016) achieved 1.10 GPa (−45%) in a steel semi-batch system [25]. The mechanical properties observed were enhanced by separating pyrolysis and oxidation, operating at reduced temperatures with minimal isothermal holding, thereby minimizing thermal stress on the fibers. This level of property retention positions the recovered fibers as suitable candidates for secondary reinforcement in thermoplastics, concrete, or non-structural composites.

### 3.4. Thermokinetics

#### 3.4.1. Model-Free Kinetics

**Figure 5:**
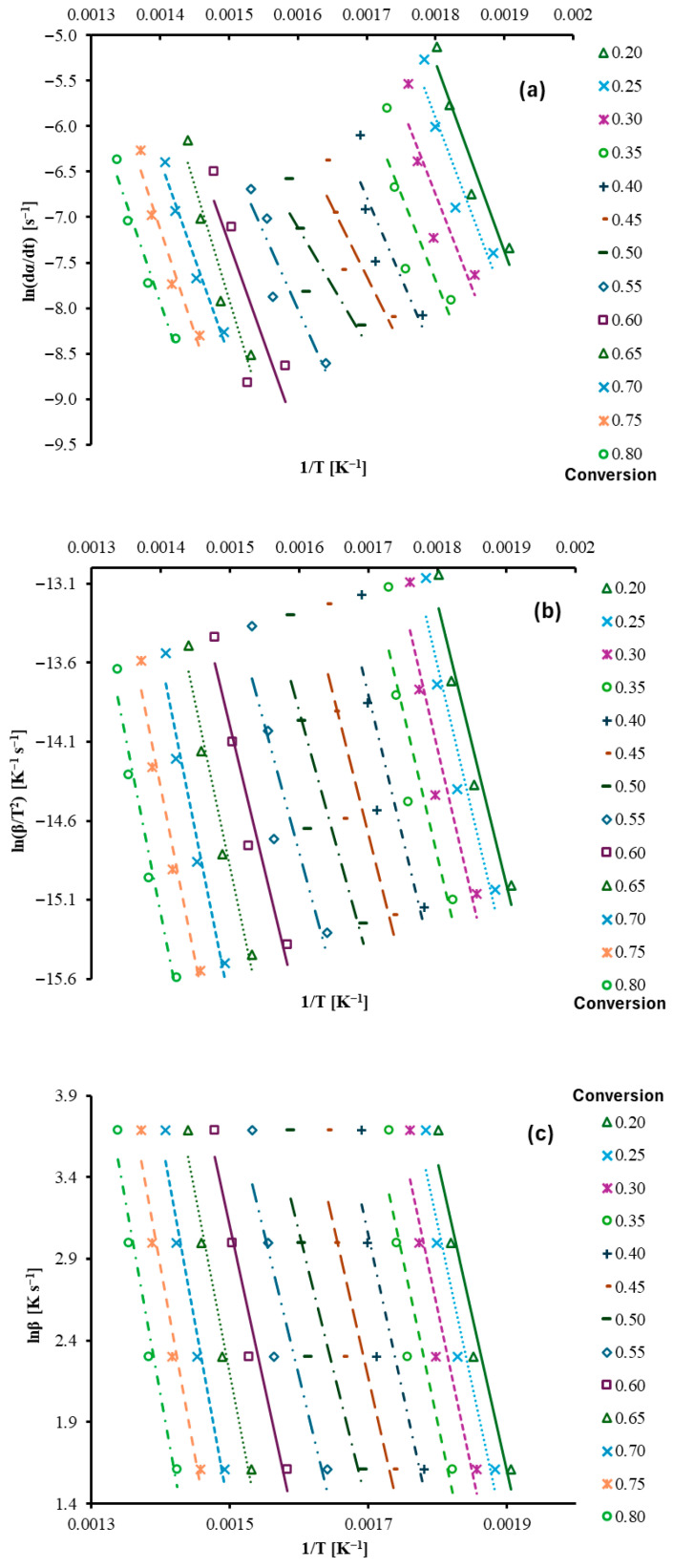
Regression lines for (**a**) Friedman, (**b**) KAS, and (**c**) OFW methods at different conversion degrees (α = 0.2–0.8).

The regression lines for the Friedman, KAS, and OFW methods are presented in Figure 5.

**Figure 6:**
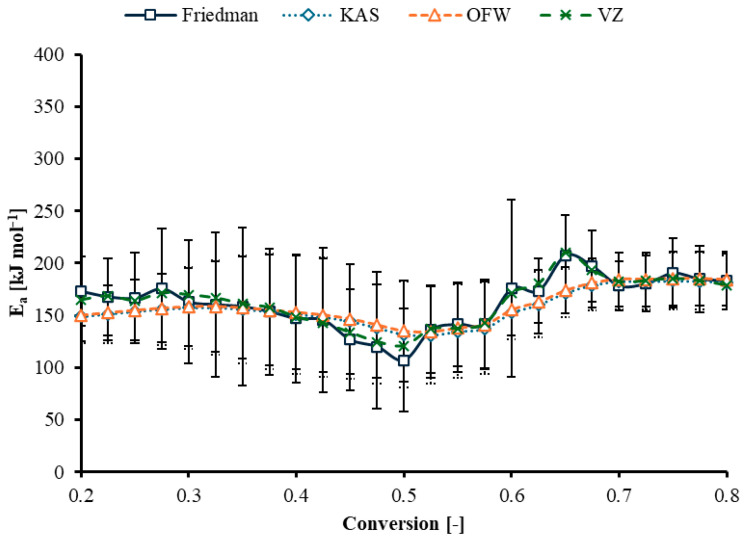
Activation energy (*E_a_*) profiles estimated from Friedman, KAS, OFW, and Advanced Vyazovkin methods.

Figure 5a shows the relationship between ln(*dα*/*dt*) and inverse temperature at different conversion levels. Each conversion has a near-linear trend. Figure 5b presents ln(*β*/*T*^2^) versus inverse temperature. Similar linear relationships are observed, and the lines shift based on conversion. This plot used the Kissinger–Akahira–Sunose method to estimate the activation energy without assuming a reaction model. The increase in slope at high conversion reflects structural resistance and the cleavage of stronger bond domains at elevated temperatures. Figure 5c shows lnβ versus inverse temperature. The resulting activation energy (*E_a_*) as a function of conversion is plotted in Figure 6. Four isoconversional kinetic models, Friedman, Kissinger–Akahira–Sunose (KAS), Ozawa–Flynn–Wall (OFW), and Vyazovkin (VZ), were applied. All four models show a similar overall pattern. *E_a_* of Friedman decreases progressively from about 172.98 kJ mol^−1^ at low conversion to about 107.06 kJ mol^−1^ at conversions of 0.50. *E_a_* then increases again after conversion at 0.55 and approaches about 183.42 kJ mol^−1^ at high conversion. Early-stage decomposition most likely involves rupture of less-ordered bonds with moderate energy requirements. Mid-conversion reflects higher accessibility of reactive structures, thereby reducing the energy barrier. Higher conversion to thermally stable structures or a char-rich residual matrix that requires higher energy for further bond scission. Ge et al. (2025) studied pyrolysis behavior in a tube furnace and estimated the kinetics of wind turbine blade materials, including epoxy resin/glass fiber (EP/GF) and thermoplastic polyurethane/carbon fiber (TPU/CF) at various mixing ratios [26]. An increase in fiber content within the EP/GF blend resulted in a more significant rise in the pyrolysis activation energy (133.69–188.94 kJ/mol) than in the TPU/CF blend (126.88–141.35 kJ/mol). Wu et al. (2023) Investigate the thermal weight loss and pyrolysis kinetics of a synthetic epoxy resin model compound, identifying a weight loss range of 300–480 °C [27]. The study shows that the pyrolysis mechanism, analyzed using the Coats–Redfern method, is diffusion-controlled, with the minimum apparent activation energy occurring at a conversion rate of 0.6.

#### 3.4.2. Combined Kinetics

**Figure 7:**
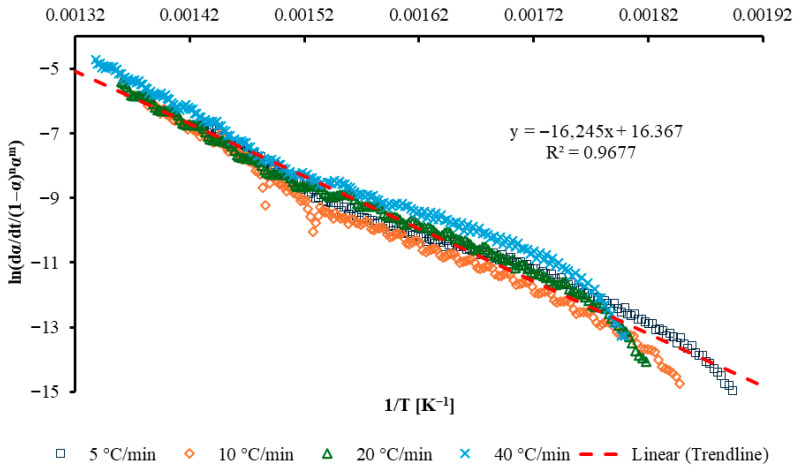
Combined kinetics plot for GFRC combustion at four different heating rates.

The combined kinetics plot using all heating rates, is shown in Figure 7.

The linear trend observed across the combined datasets indicates that thermal decomposition follows a temperature-dependent Arrhenius behavior over the examined conversion range. The activation energy *E_a_* is 134 ± 4.8 kJ/mol, the reaction order *n* is 1.79 ± 0.23, and the parameter *m* is −5.5 ± 0.23, while ln(cA) is 16.2 ± 1.1 s^−1^.

### 3.5. Machine Learning Prediction

**Figure 8:**
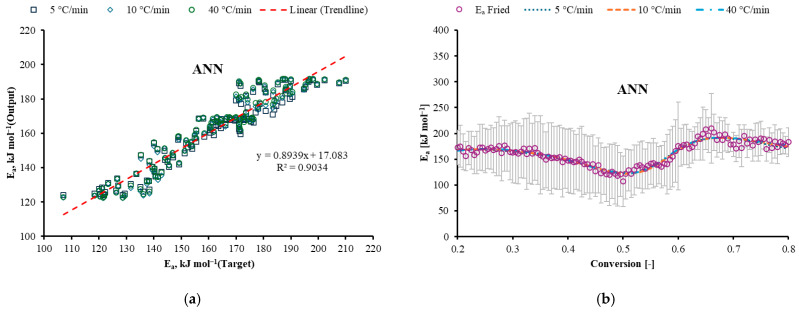

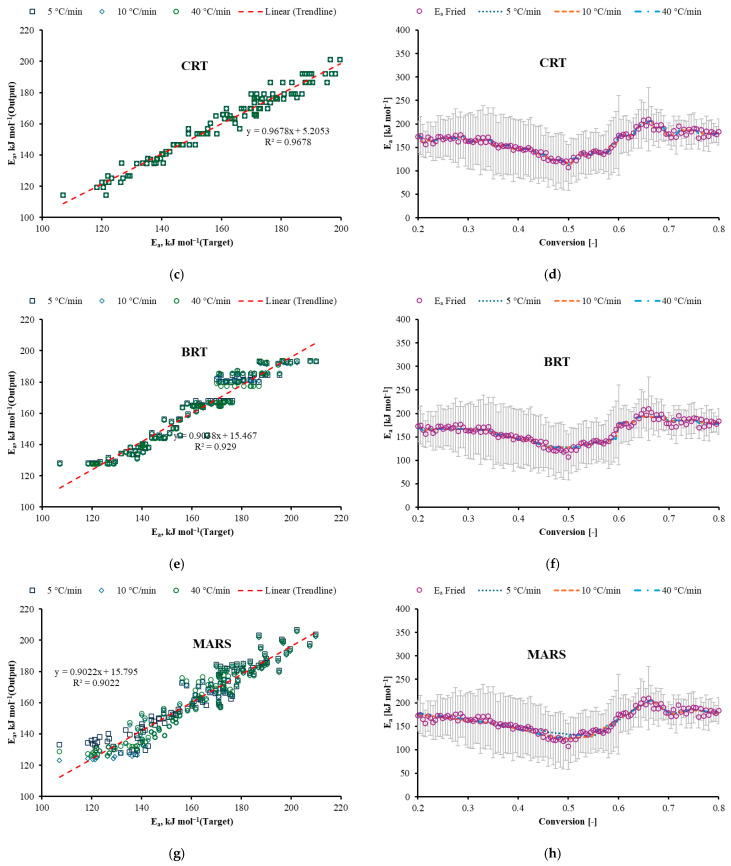
(**a**,**c**,**e**,**g**) Correlation between target and ANN, CRT, BRT, and MARS model output for *E_a_*. (**b**,**d**,**f**,**h**) Prediction of *E_a_* during conversion at 5, 10, and 40 °C/min for the corresponding models.

The performance of the four machine learning models (MLP, CRT, BRT, and MARS) in predicting the Friedman-derived activation energy is compared in Figure 8. Figure 8a,c,e,g show the correlation between the target (experimental) *E_a_* and the model-predicted *E_a_* for each algorithm. All models demonstrated a strong linear correlation. The CRT model, in particular, achieved a high coefficient of determination (R^2^ = 0.9678), indicating its excellent predictive accuracy for this specific dataset. Figure 8b,d,f,h display the prediction of *E_a_* as a function of conversion at heating rates of 5, 10, and 40 °C/min for the corresponding models. The plots show how each ML algorithm captures the complex, non-linear trend of increasing activation energy with conversion. The predicted profiles from all models, especially CRT and BRT, closely follow the experimental Friedman data, successfully replicating the multi-step nature of the GFRC combustion process. This demonstrates the robustness of these data-driven approaches in modeling intricate kinetic behavior where traditional model-fitting methods struggle.

The performance metrics for all models are summarized in Table 4. While all models showed good predictive capability (R^2^ > 0.9), CRT achieved the optimal balance between accuracy and computational efficiency, with the highest R^2^ (0.968) and lowest RMSE (4.07 kJ/mol) among the tree-based methods.

**Figure 9:**
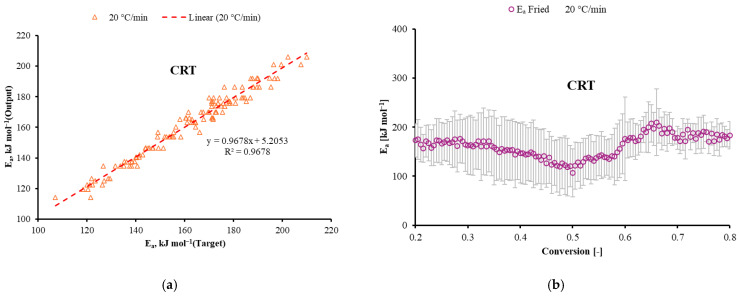
(**a**) Correlation between target and CRT model output for *E_a_* at 20 °C/min. (**b**) Prediction of *E_a_* during conversion at 20 °C/min using the CRT model.

The superior performance of CRT can be attributed to its ability to handle discontinuous relationships in the conversion-dependent activation energy profile (Figure 9). The increasing *E_a_* trend reflects the transition from initial polymer chain scission (lower *E_a_*) to subsequent oxidation of carbonaceous char and decomposition of the fiber–matrix interface (higher *E_a_*). CRT effectively captured these mechanistic transitions through its decision boundaries, while maintaining computational efficiency compared to the more complex MLP and MARS models. The CRT machine learning model was deployed to predict Friedman-derived *E_a_* values. Figure 6 shows the correlation between the target (experimental) and model-predicted *E_a_* values for the 20 °C/min dataset which was not included in the earlier training, testing, and validation. The CRT model achieved an R^2^ of 0.9678, demonstrating its strong predictive capability for the non-linear combustion kinetics of GFRC. Shahroodi et al. (2026) introduced an integrated experimental and computational framework that employs Artificial Neural Networks (ANN), achieving high predictive accuracy (R^2^ > 0.85) to optimize and forecast the tensile properties of glass fiber-reinforced recycled polypropylene (GF-rPP) [28]. Another recent study by Chen et al. (2025) applied an Artificial Neural Network (ANN) as a data-driven non-parametric model to jointly predict multiple outputs related to the pyrolysis of glass fiber/epoxy resin composites [29].

## 4. Conclusions

This work confirms that clean glass fiber recovery from wind-blade GFRP is possible using significantly milder thermochemical windows than those commonly reported in the literature, which often operate at 500 °C or higher with long isothermal exposure. The explicit separation of polymer volatilization during pyrolysis and char removal during oxidation allowed us to systematically decouple the thermal functions and identify the minimum required exposure for each stage. The experimental results show that a 0 min pyrolysis at 425 °C, followed by a 0 min isothermal dwell, is sufficient to initiate matrix removal without damaging the fibers, and that a 15 min oxidation at 475 °C is enough to achieve full visual cleanliness of the fibers. Notably, the recovered fibers preserved a tensile strength of 1.52 GPa and a Young’s modulus of 62 GPa, which reflect a reduction of only 24% and 12%, respectively, relative to the pristine E-glass. The observed performance is superior to conventional pyrolysis methods, thereby supporting the feasibility of rGFs in applications with moderate performance requirements, such as thermoplastic composites or fiber-reinforced concrete. Combined kinetic evaluation confirmed that the activation energy varies strongly with conversion due to multiple reaction steps, and machine learning methods accurately predicted this trend. The moderate energy requirement and short thermal residence time present a realistic pathway for industrial retrofitting and scale-up, as the thermal duty becomes manageable and the process becomes compatible with continuous or semi-continuous ovens. The scientific implication is that fiber retention quality and matrix removal can be balanced through conservative process design if pyrolysis and oxidation are clearly separated instead of forcing both into a single thermal event. This work also highlights that thermal history, rather than absolute peak temperature, controls the balance between fiber integrity and matrix decomposition. The practical implication is that wind-blade waste can be treated using energy-efficient thermochemical processes, thereby reducing the greenhouse gas burden relative to conventional disposal or higher-temperature oxidative treatment. Future research should now quantify the mechanical properties of the recovered fibers, define re-sizing protocols that restore adhesion, and link fiber quality classes to specific secondary markets, including thermoplastic compounding and cementitious composites. Industrial translation would benefit from process integration with gas and oil valorization, and from standardization of fiber quality grades. The set of conservative windows identified here can therefore serve as an engineering baseline for industrial thermochemical recovery plants targeting GFRP.

## Figures and Tables

**Table 1:** Glass fiber-reinforced composite thermochemical recycling using pyrolysis.

Study (Year)	Feedstock/Context	Pyrolysis Process and Conditions	Key Findings	Challenges
Naqvi et al. (2018) [3]	CFRC/GFRC from automotive, wind, aerospace	Two-step pyrolysis, 300–700 °C, lab scale	Moderate energy use (30 MJ/kg), viable for fiber and fuel recovery, promising for the circular economy	Optimization and commercialization challenges, fiber quality, and energy use
Caballero et al. (2025) [10]	Glass fiber-reinforced polyamide (auto waste)	Two-step: 500 °C pyrolysis + 900 °C thermal cracking	Improved gas quality, reduced global warming impact, better LCA than conventional pyrolysis	Pre-existing fiber damage limits quality
Karuppannan Gopalraj & Kärki (2020) [11]	General review (CF/GF composites)	Mechanical, thermal (pyrolysis/fluidized-bed), chemical	Pyrolysis preserves fiber better than combustion, up to 20% fiber reuse	Environmental/ economic trade-offs, fiber property loss
Yousef et al. (2021) [7]	GFRC panels (epoxy)	TG-FTIR, 256–500 °C	Three-stage decomposition, major volatiles: phenol, benzene; activation energy 165–193 kJ/mol	Mechanical pre-treatment affects kinetics
Miyazawa & Wajima (2021) [12]	Waste GFRP	Pyrolysis with KOH, inert atmosphere	Promotes gasification, converts glass fiber to soluble silicate	High temp/cost, residue handling
Yousef et al. (2022) [13]	GFRP + nanofillers	Catalytic pyrolysis (ZSM-5), 5–30 °C/min	Lower activation energy, higher volatile yield	Filler effects, catalyst cost
Takaaki & Miyagawa (2022) [14]	Waste GFRP	Pyrolysis with NaOH, microwave heating	75% energy reduction vs. conventional, efficient silica extraction	Microwave scale-up, residue handling
Serras-Malillos et al. (2023) [8]	EoL GF polyester	900 °C, two-stage	Maximized syngas, minimized hazardous liquids	High temp, process scale
Rafay et al. (2024) [9]	EoL GFRP panels	Pyrolysis + partial oxidation + hot alkaline etching	200% fiber strength increase vs. pyrolysis alone	Process complexity, short etching cycles

**Table 2:** Process conditions for the experimentation [16].

Platin		Post-Pyrolysis (Air)	
Temperature (°C)	Time (min)	Temperature (°C)	Time (min)
**Effect of temperature on pyrolysis**			
**350**	30	-	-
**400**	30	-	-
**425**	30	-	-
**450**	30	-	-
**Effect of residence time on pyrolysis**			
**425**	0	-	-
**425**	15	-	-
**425**	30	-	-
**Effect of temperature on post-pyrolysis**			
**425**	30	400	30
**425**	30	450	30
**425**	30	475	30
**425**	30	500	30
**Effect of residence time on post-pyrolysis**			
**425**	0	475	0
**425**	0	475	15
**425**	0	475	30

**Table 3:** Mechanical properties of recovered glass fibers.

Reference	Reactor Type	Tensile Strength (GPa)	Young’s Modulus (GPa)
Giorgini, Leonardi et al. (2016) [24]	Fixed-bed reactor (pyrolysis)	1.02 (−50%)	53 (−25%)
Onwudili, Miskolczi et al. (2016) [25]	Steel semi-batch reactor (pyrolysis)	1.10 (−45%)	60 (−15%)
This study	Batch-type pyrolyzer (two-step mild thermochemical)	1.52 (−24%)	62 (−12%)

**Table 4:** Performance comparison of machine learning models.

Model	AME	MSE	RMSE	R^2^	Valid N
**ANN**	5.647	49.780	7.055	0.903	363
**CRT**	3.267	16.588	4.073	0.968	363
**BRT**	4.662	36.870	6.072	0.928	363
**MARS**	5.679	50.336	7.095	0.902	363

## Data Availability

The original contributions presented in this study are included in the article. Further inquiries can be directed to the corresponding authors.
